# Supplementary material for: Considerations for ensuring safety during telerehabilitation of people with stroke. A protocol for a scoping review
Source: PLoS One. 2023 Jan 6;18(1):e0276354. doi: 10.1371/journal.pone.0276354 (PMC9821787; doi:10.1371/journal.pone.0276354)
Supplement: Attachment — Submitted filename: Response to Reviewers.docx [file pone.0276354.s002.docx]

**Response to Reviewers**

| **Comments** | **Responses** |
| --- | --- |
| Line: 68: Why only a small or moderate impact on quality of life? | The effect is small to moderate due to the values of the 95% CI limits of the standardized mean difference estimated by the study of Ali et al. Specific numerical results were added in lines 69 and 70 of the revised manuscript. |
| Some sentences are confusing in the wording, e.g. line 70-72. I think also strategies for telerehabilitation had to be adapted? Or does it mean a the implementation of a new concept like telerehabilitation is a positive effect of the pandemic? Then I would describe it in a more positive manner. | The idea was rephrased to give it a more "positive" look.  In lines 73 to 74 the following sentence was added: “…however, strategies such as telerehabilitation were implemented, which contributed to continued service delivery.” |
| Why do you not refer to other diseases with positive impacts due to telerehabilitation to support your research interests? (e.g. Piotrowicz E. et al. doi: 10.1001/jamacardio.2019.5006. | The reference suggested by the reviewer and others have been added. |
| Also I miss, the different kinds of telerehabilitatio, e.g. telerehabilitation in mobility, or speech training | Four functions were added where telerehabilitation could have an impact on people with stroke.  The following sentence was added to lines 81 to 82: “Telerehabilitation in stroke patients can be used to deliver interventions aimed at improving cognitive [26], physical [22,27], speech [28] and swallowing function [29].” |
| Please write out the abbreviation PCC (line 112) | Abbreviation added on line 117 of the revised manuscript. |
| Line 130: Will they distinguish between the different evidence levels of the included studies? | The level of evidence is reported according to the Oxford scale. This information was added to table 2. |
| Line 140: Please describe necassary qualification of reviewers | The following idea was added to the revised manuscript: “Reviewers involved in study selection and data extraction should have clinical experience in the care of stroke patients through tele-rehabilitation, as well as training in conducting evidence synthesis studies. A novice reviewer will always be paired with a senior reviewer.” |
| Line 142: Please describe the benefit of using the Rayyan® app | The following idea was added to the revised manuscript: “This tool improves the efficiency of the study selection process both in the title-abstract and full-text screening phase, eliminates duplicate records, facilitates the construction of the study selection diagram and helps to resolve disagreements.” |
| Line 158: Do not repeat the sentence exactly of line 144-45. Maybe you can write this as overall method in case of disagreements. | Both sentences were deleted, and one was added.  The following idea was added to the revised manuscript: “For both the selection and extraction phases, disagreements will be resolved by consensus. If this is not achieved a third reviewer will make the final decision.” |
| Is it planned to contact study investigator in case of missing or confirming information | No information will be required from the authors of the studies in the cases indicated, as this review also seeks to determine the quality of the reporting of the studies.  The following idea was added to the revised manuscript: “In case the information presented by the studies is unclear or missing, the authors of the studies will not be contacted, as this scoping review also aims to assess the completeness of the reporting of the studies.” |
| Which tiime frame of the analysis will be covered? | In the "Study design" sub-section of the eligibility criteria, it is reported that studies will not be restricted for inclusion based on date of publication. |
| Please include a bias or evidence grade information of the study in table 2 depending on the kind of study and also of number of included patients in the study. | The risk of bias and certainty of evidence is assessed when we want to determine how much we can believe the results of studies. In addition, the certainty of evidence is determined for each outcome reported by the study, and not for single studies.  Taking into account the above two points, associated with the fact that scoping reviews do not seek to determine the effectiveness of interventions (that is the task of systematic reviews and meta-analyses) and that the objective of our review is to determine the measures implemented to prevent adverse events during tele-rehabilitation of people with stroke, the assessment of risk of bias and certainty of evidence does not apply to our study. |
| The authors stated that "The search was not limited by publication date, publication status, or the language of the". Future tense should be preferred for the whole manuscript. | The sentence was changed to the future tense. |
| Also, authors should give more details on Rayyan software | The following idea was added to the revised manuscript: “This tool improves the efficiency of the study selection process both in the title-abstract and full-text screening phase, eliminates duplicate records, facilitates the construction of the study selection diagram and helps to resolve disagreements.” |
